# *Orthosiphon stamineus* Standardized Extract Reverses Streptozotocin-Induced Alzheimer’s Disease-Like Condition in a Rat Model

**DOI:** 10.3390/biomedicines8050104

**Published:** 2020-04-30

**Authors:** Thaarvena Retinasamy, Mohd. Farooq Shaikh, Yatinesh Kumari, Syafiq Asnawi Zainal Abidin, Iekhsan Othman

**Affiliations:** 1 Neuropharmacology Research Strength, Jeffrey Cheah School of Medicine and Health Sciences, Monash University Malaysia, Bandar Sunway, Selangor 47500, Malaysia; thaarvena@gmail.com (T.R.); yatinesh.kumari@monash.edu (Y.K.); syafiq.asnawi@monash.edu (S.A.Z.A.); 2 Liquid Chromatography Mass Spectrometry (LC-MS) Platform, Jeffrey Cheah School of Medicine and Health Sciences, Monash University Malaysia, Bandar Sunway, Selangor 47500, Malaysia

**Keywords:** Alzheimer’s disease, cognitive function, streptozotocin, *Orthosiphon stamineus*, oxidative stress

## Abstract

Alzheimer’s disease (AD) is a chronic neurodegenerative brain disease that is characterized by impairment in cognitive functioning as well as the presence of intraneuronal neurofibrillary tangles (NFTs) and extracellular senile plaques. There is a growing interest in the potential of phytochemicals to improve memory, learning, and general cognitive abilities. The Malaysian herb *Orthosiphon stamineus* is a traditional remedy that possesses anti-inflammatory, anti-oxidant, and free-radical scavenging abilities, all of which are known to protect against AD. Previous studies have reported that intracerebroventricular (ICV) administration of streptozotocin (STZ) mimics a condition similar to that observed in AD. This experiment thus aimed to explore if an ethanolic leaf extract of *O. stamineus* has the potential to be a novel treatment for AD in a rat model and can reverse the STZ- induced learning and memory dysfunction. The results of this study indicate that *O. stamineus* has the potential to be potentially effective against AD-like condition, as both behavioral models employed in this study was observed to be able to reverse memory impairment. Treatment with the extract was able to decrease the up-regulated expression levels of amyloid precursor protein (APP), microtubule associated protein tau (MAPT), Nuclear factor kappa-light-chain-enhancer of activated B cells (NFᴋB), glycogen synthase kinase 3 alpha (GSK3α), and glycogen synthase kinase 3 beta (GSK3β) genes indicating the extract’s neuroprotective ability. These research findings suggest that the *O. stamineus* ethanolic extract demonstrated an improved effect on memory, and hence, could serve as a potential therapeutic target for the treatment of neurodegenerative diseases such as AD.

## 1. Introduction

Alzheimer’s disease (AD) is an age-related brain disease and one of the most common types of dementia. AD is characterized by chronic and progressive neurodegeneration that triggers advanced cognitive impairment, ultimately leading to death [1,2]. The pervasiveness of this disease is expected to quadruple from 26.6 million cases (1 in 253 people) to 1 in 85 people living with the disease by 2050 [3]. Those diagnosed with this disease are unable to encode new memories, which in turn damages both declarative and non-declarative memory, thus gradually reducing the ability for reasoning, abstraction, and language [4]. Elderly people are most predisposed to developing AD and the risk increases with age. There are namely two forms of the disease, sporadic (SAD) and familial (FAD). It has been predicted that the pervasiveness of SAD is projected to be increased to 131.5 million in 2050 which in turn could lead to a serious socio-economic burden [5]. 

AD is largely characterized by the presence of intraneuronal neurofibrillary tangles (NFTs) and extracellular senile plaques together with neurodegeneration in the brain [2,6]. The key etiological component of AD includes a causative protein, amyloid β protein, whereby the aggregation and deposition of the amyloid β protein activate an inflammatory immune reaction that in turn obliterates the brain neurons [7]. The synthesis of amyloid is regulated by the secretase enzyme. Thus, it can be further said that the damage to the amyloid β peptide cerebral clearance causes an abnormal increase in its brain level during the late onset of AD, which in turn accounts for most of the AD cases [8]. Another key hallmark of AD is the decrease in the production of the neurotransmitter acetylcholine, which is vital in controlling various memory-related functions [9]. AD predominantly affects cholinergic neurons in the cerebral cortex of the brain, whereby the neuronal activity is largely controlled by the neurotransmitter “acetylcholine” [10,11]. In AD, memory decline occurs because the enzyme that produces acetylcholine becomes defective resulting in a shortage of this neurotransmitter at the neuronal synapse [11]. Additionally, recent studies have shown that neuronal degeneration linked with AD has been triggered largely by neuroinflammation, oxidative stress, neurotransmitter imbalance, and neurotoxicity [5,12,13].

Streptozotocin (STZ) is a glucosamine-nitrosourea compound that produces a cytotoxic agent that particularly affects the β cells in the pancreatic islet, impairing the brain biochemistry and cholinergic transmission, as well as increasing the generation of free radicals [14,15,16]. Intracerebroventricular administration of STZ has been shown to resemble the similar neuropathology and biochemical alterations observed during an AD condition thus resulting in STZ-induced models playing a vital role in the pathophysiology of sporadic Alzheimer’s.

In recent times, there has been a growing interest in using complementary therapy and phytochemicals from medicinal herbs to enhance the quality of life and prevent therapy-induced side-effects, particularly in using phytochemicals from medicinal herbs, as they possess anti-inflammatory and antioxidant activities that may potentially hinder neurodegeneration and improve memory and cognitive functioning [17]. *Orthosiphon stamineus* Benth. (Lamiaceae) is a medicinal herb that is extensively distributed in South East Asia. Various in vitro and in vivo models have addressed the presence of different types of phytochemicals in this plant-like flavonoids, terpenoids, and essential oils. Earlier studies have demonstrated that *O. stamineus* (OS) leaves extracts to possess strong antioxidant, anti-inflammatory, and anti-bacterial properties, with more than 20 phenolic compounds, two flavonol glycosides, nine lipophilic flavones, and nine caffeic acid derivatives, such as rosmarinic acid and 2,3-di-caffeoyl tartaric acid and nitric oxide inhibitory isopimarane-diterpenes [18,19,20,21]. For instance, Rosmarinic acid, a major flavonoid component of *O. stamineus* has been shown to have various pharmacological properties. Flavonoids, which are the principal group of polyphenols, are also reported to be efficacious in decreasing oxidative stress and are said to promote various physiological benefits, particularly in learning and memory, scavenging free radicals and improving cognition [6,22]. Besides that, standardized ethanolic extract of *O. stamineus* was also found to be able to reverse age-related deficits in short-term memory as well as prevent and reduce the rate of neurodegeneration [23]. Moreover, in vitro studies have demonstrated that *O. stamineus* enhanced H_2_O_2_ induced oxidative stress by antioxidant mechanisms in SH-SY5Y human neuroblastoma cells [24].

Therefore, since the OS extract has been observed to demonstrate strong antioxidant and anti-inflammatory properties, these reports on the OS extract further support its neuroprotective potential in combating neurodegenerative diseases such as AD. Based on the activity profile of OS extract, it can be hypothesized that OS could serve to halt or modify intricate neurodegenerative diseases such as AD. Thus, the aim of this study was to investigate the protective potential of OS against STZ-induced AD-like condition, using two established behavioral paradigms for learning and memory as well as to observe the hippocampal alterations associated. Since preliminary studies have demonstrated a neuroprotective as well as cholinesterase inhibitory effect; hence, it is hypothesized that *O. stamineus* can be established as an effective and safer potential therapeutic agent to combat cognitive alterations in AD. 

## 2. Materials and Methods

### 2.1. Plant Extract Standardization of Orthosiphon stamineus 50% Ethanolic Extract

The plant material was collected from NatureCeuticals HiTech Plantation, Jalan Kampung Binjai, Kampung Binjai, 11960 Batu Maung, Pulau Pinang, Malaysia (5°16’59.4” N 100°15′30.3” E) and the sample was authenticated and deposited at the Herbarium of School of Biology, Universiti Sains Malaysia with voucher #11009. The 50% ethanolic OS extract was procured from NatureCeuticals Sendirian Berhad, Kedah DA, Malaysia. The standardized extract from leaves of Orthosiphon stamineus was prepared under a good manufacturing practice (GMP)-based environment using Digmaz technology by Natureceuticals Sdn. Bhd., Malaysia. The 50% ethanolic *O. stamineus* extract was standardized by Natureceuticals Sdn. Bhd., Malaysia. As per their standardization report, the standardization of the extract was carried out against four bioactive standard markers; rosmarinic acid (RA), sinensetin (SIN), eupatorin (EUP), and 3′hydroxy-5,6,7,4′-tetramethoxyflavone (TMF). 

### 2.2. LC-MS Analysis

The MS analysis was performed on an Agilent UHPLC 1290 Infinity system coupled to Agilent quadrupole-time-of-flight 6520 mass spectrometer with dual ESI source. The sample was loaded on a C18 column (XDB-C18 Agilent Zorbax Eclipse, narrow-bore 2.1 × 150 mm, 3.5 micron, P/N: 930990–902). The thermostat temperature was maintained at 25 °C and the auto-sampler temperature was set at 4 °C. The mobile phases used were 0.1% formic acid in water (A) and 0.1% formic acid in acetonitrile (B). The sample was eluted by increasing the gradient of buffer B from 5–95% over 25 min at a flow rate of 0.5 mL/min. The injection volume was 1.0 µL. MS analysis scan was carried out in a range of m/z 100–1000 employing electrospray ion source in the positive ionization mode. Nitrogen gas flow rate and drying gas were set at 25 L/h and 600 L/h, respectively. Drying gas temperature was set at 350 °C. The fragmentation voltage was optimized to 125 v, while the capillary voltage for analysis was 3500 v.

### 2.3. Animals

Locally-bred adult male Sprague Dawley (SD) rats weighing between 200–300 g were acquired from the animal facility of Jeffrey Cheah School of Medicine and Health Sciences, Monash University Malaysia. The rats were maintained under standard husbandry conditions (12:12 h light/dark cycle, at controlled room temperature (22 ± 2 °C), stress-free, water ad libitum, standard diet, and sanitary conditions). The experiment protocols were approved and conducted according to the approval of the Animal Ethics Committee Monash University, Animal Research Platform (MARP/2016/028).

### 2.4. Intracerebroventricular (ICV) Infusion of Streptozotocin

Streptozotocin (STZ) was injected ICV bilaterally at a dose of 3 mg/kg as described previously [15]. Briefly, the rats were firstly anesthetized using a combination of ketamine hydrochloride (75 mg/kg, intraperitoneally (i.p.)) and xylazine (10 mg/kg, i.p.). The head was positioned and fixed on the stereotaxic frame. A midline sagittal incision was done on the scalp and a burr hole was drilled through the skull on both sides over the lateral ventricles. The coordinates employed were: 0.8 mm posterior to bregma, 1.5 mm lateral to the sagittal suture, and 3.6 mm beneath the surface of the brain [25]. An injection cannula was lowered very slowly into the lateral ventricles to deliver STZ (3 mg/kg, 10 µL/injection site) or saline (10 µL/injection site) through the skull holes. STZ was prepared freshly before each injection. The injection cannula was connected to a Hamilton syringe and the injection was done using a micro-injector unit. The cannula was left in situ for a further 5 min following the injection to allow passive diffusion from the cannula tip and to minimize spread into the injection tract. The cannula was then removed slowly from the scalp and the cut skin was closed with sutures. Following the surgery, postoperative care was made by applying the betadine povidone-iodine solution on the wound. The rats were also placed on thermal sheets to maintain body temperature and were kept under close observation for the next four days. 

### 2.5. Experimental Design

Before the experiment, animals were acclimatized to the surroundings and were handled for one week to reduce the stress. The animals were then randomly divided into five groups (n = 8/group) as described below. The ethanolic extract of OS was dissolved in distilled water before administration.

Group 1: Sham-control (Saline)

Group 2: Negative control (Saline + STZ; 3 mg/kg)

Group 3: STZ (3 mg/kg) + Orthosiphon stamineus Low dose (50 mg/kg OS)

Group 4: STZ (3 mg/kg) + Orthosiphon stamineus Medium dose (100 mg/kg OS)

Group 5: STZ (3 mg/kg) + Orthosiphon stamineus High dose (200 mg/kg OS)

**Figure 1:**
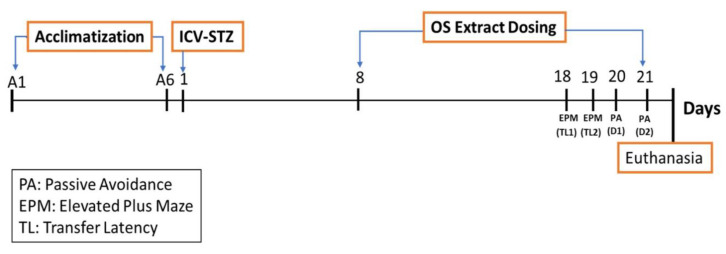
Schematic representation of the experimental flow. ICV-STZ: intracerebroventricular administration of streptozotocin; OS: *Orthosiphon stamineus*.

After seven days of acclimatization period, the animals were subjected to ICV injection. Group 1 rats were sham-operated, where only the surgery was done, and the brain was injected with saline, whereas groups 2–5 received STZ (3 mg/kg, single injection bilaterally). One week after the ICV-STZ injection, the rats were treated with OS extract through oral dosing using oral gavage for 10 days before being subjected to a series of behavioral studies. The behavioral parameters were conducted on day 18, where elevated plus maze test was conducted on the day (18 and 19) and a passive avoidance test was conducted on the day (20 and 21). At the end of the study, the animals were sacrificed, and their brains were isolated for gene expression analysis. The treatment schedule is presented in Figure 1.

### 2.6. Elevated Plus Maze (EPM)

The elevated plus-maze test was employed to evaluate acquisition and retention memory following the procedure previously described [15]. Briefly, after being treated with the OS extract, the rats were put on to the end of the open arm, facing away from the central platform. With the help of the stopwatch, the transfer latency (TL_1_) was noted, i.e., the time taken by a rat with all its four legs to move into any one of the enclosed arms. If the rat failed to enter any one of the enclosed arms within 90 s, it was gently pushed into one of the two enclosed arms and the TL was assigned as 90 s. The rat was allowed to explore the maze for the next 10 s and then returned to its home cage. The maze was cleaned with 70% ethanol between runs to minimize scent trails. The retention test phase was carried out 24 h after the training session to assess memory, whereby a decrease in time latency (TL_2_) during the test session was deemed as an index of memory improvement. The transfer latency was expressed as an inflexion ratio, calculated using the formula:

(1) $$\mathrm{IR}=\frac{\left(L1-L0\right)}{L0}$$

L_0_: Initial TL (s) on the 1st day and 

L_1_: TL (s) on the 2nd day. 

### 2.7. Passive Avoidance (PA)

A step-through passive avoidance (PA) test was carried out to measure memory retention deficit using the Passive Avoidance Box (Panlab, Harvard Apparatus) following the method previously used [26,27]. Briefly, during the acquisition trial, each rat was placed in the light chamber. Following the 60 s of habituation, the guillotine door separating the light and dark chamber was opened and the initial latency time for the rat to enter the dark chamber was recorded. The rats with the initial latency time of more than 60 s were excluded from the study. Once the rat entered the dark chamber, the guillotine door was closed and an electric foot shock (75 V, 0.2 mA, 50 Hz) was delivered to the floor grids for 3 s. Five seconds later, the rat was removed from the dark chamber and returned to its home cage. After 24 h, the retention latency was measured in the same way as the acquisition trial, but the foot shock was not delivered, and the latency time was recorded to a maximum of 300 s. 

### 2.8. Gene Expression

Total RNA from the rat brain’s hippocampal and pre-frontal cortical region was extracted following the method employed by [28], with some minor modifications. The single-step method, phenol-chloroform extraction, and Trizol reagent (Invitrogen) were used to isolate the total RNA from both the pre-frontal cortical and hippocampal regions. Briefly, the tissues were homogenized in 200 µL of Trizol solution. The mixture was then extracted using chloroform and centrifuged at 135,000 rpm at 4 °C. The alcohol was removed, and the pellet was washed twice with 70% ethanol and resuspended in 20 µL of RNase free water. RNA concentration was determined by reading absorbance at 260 nm using Nanodrop. A 500 ng amount of total RNA was reverse transcribed to synthesize cDNA using the Quantitect^®^ Reverse Transcription Kit according to the manufacturer’s protocol. Then, the mRNA expression of genes encoding amyloid precursor protein (APP), Microtubule Associated Protein Tau (MAPT), Nuclear factor kappa-light-chain-enhancer of activated B (NFᴋB), Glycogen synthase kinase-3 alpha (GSK3α), Glycogen synthase kinase-3 beta (GSK3β), and IMPDH2 in the hippocampus was measured via real-time PCR using the StepOne Real-Time PCR system. Subsequently, the cDNA from the reverse transcription reaction was subjected to Real-Time PCR using QuantiNova™ SYBR^®^ Green PCR kit according to the manufacturer’s protocol. The comparative threshold (C_T_) cycle method was used to normalize the content of the cDNA samples, which consists of the normalization of the number of target gene copies versus the endogenous reference gene, IMPDH2.

### 2.9. Statistical Analysis

All findings were expressed as mean ± standard error of the mean (SEM). The data were analyzed using one-way analysis of variance (ANOVA) followed by Dunnett’s tests. All the experimental groups were compared with group 2, the STZ (3 mg/kg) only group, and the *p*-values of ^∗^ *p* < 0.05, ^∗∗^ *p* < 0.01, and ^∗∗∗^ *p* < 0.001 were considered to be statistically significant. 

## 3. Results

### 3.1. Characterization of O. stamineus Ethanolic Extract

The 50% ethanolic extract was found to contain four markers, namely, RA, SIN, EUP, where the amounts of TMF were present in a very low amount as compared to the other markers, while the RA was present in abundance. 

### 3.2. LC-MS Analysis

**Figure 2:**
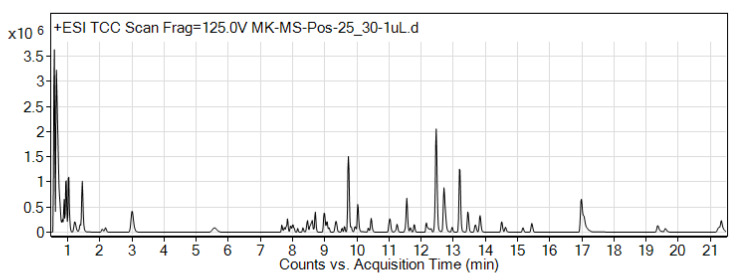
Total Compound Chromatogram (TCC) of *Orthosiphon stamineus* extract..

Identification of small-molecule contents in OS extract were detected using LC-MS analysis. A positive ionization mode was utilized for the tentative identification of the compounds. The total compound chromatogram (TCC) of the extract demonstrated different peaks as shown in Figure 2. The LC-MS analysis of the OS extract identified a total of 87 different compounds (Table 1) belonging to various groups, e.g., phenols, flavonoids, amino acids, coumarins, carboxylic acid, sesquiterpenoid, nucleoside, quinone, and cinnamic acid. Flavonoids were identified as the major compound present in the extract, such as (R)-O-(3,4-Dihydroxycinnamoyl)-3-(3,4-dihydroxy phenyl)lactic acid, Quercetagetin 4′-methyl ether 7-(6-(E)-caffeylglucoside), Luteolin 7-rhamnosyl(1→6)galactoside, Prodelphinidin A1, 6-Hydroxyluteolin 7-rhamnoside, Xanthochymuside, and Iriskumaonin. 

### 3.3. Effect of OS Extract on Memory Performance in EPM and PA Task in ICV-STZ Infused Rats 

**Figure 3:**
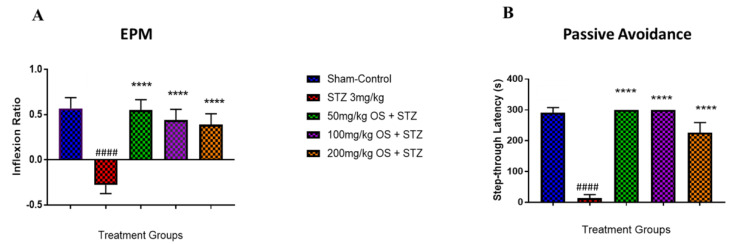
Behavioral analysis for elevated plus maze (EPM) and passive avoidance (PA). (**A**) represents the graph plot for the inflection ratio; (**B**) represents the graph plot for the step-through latency in PA. The behavioral analysis for the treatment groups (**A**,**B**) was compared to the negative group (3 mg/kg STZ) and the negative group (3 mg/kg STZ) was compared to the control group. Data are expressed as Mean ± SEM, *n* = 8 and statistical analysis by one-way ANOVA followed by Dunnett test **** *p* < 0.0001, ^####^ *p <* 0.0001.

Both the EPM and PA task was carried out to assess spatial long-term memory retention. In the EPM task, as observed in Figure 3A, the negative (only STZ-induced) group demonstrated a notable decrease in inflexion ratio whereas the OS treated groups demonstrated a significant increase in inflexion ratio when compared to the negative group. In the PA task, memory performance was assessed by determining the latencies to enter the dark (shock-paired) compartment during the post-24-h retention trial. All the groups did not demonstrate any differences in latency during the learning trial (data not shown), signifying that all rats showed similar responses to the testing environment and electric shocks. On the other hand, the retention test that was performed 24 h following the initial training demonstrated a significant decrease in step-through latency in the negative (only STZ-induced) group as compared to the sham-operated and the other treated groups as depicted in Figure 3B. However, when the STZ-induced rats were treated with all the three doses (50, 100, and 200 mg/kg) of OS extract, a significant increase in step-through latency was observed, indicating improved memory retention. Based on these results obtained, it can be said that OS extract does improve memory retention. 

### 3.4. Effect of OS Extract on the Gene Expression in the Rat Hippocampal and Prefrontal Cortical Region 

**Figure 4:**
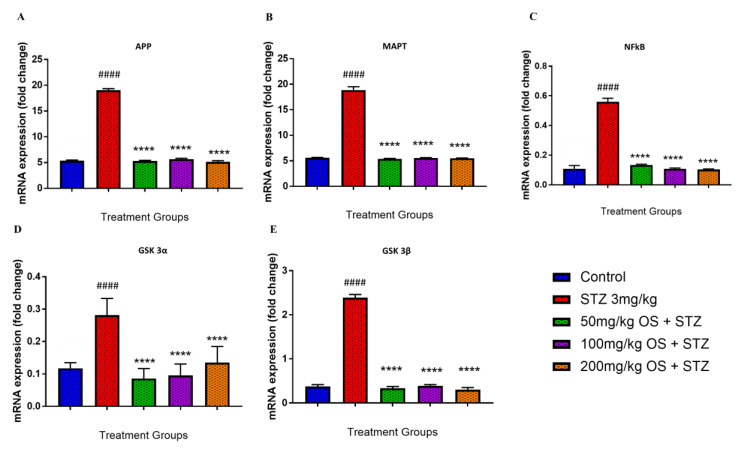
Gene expression in the rat hippocampi determined by real time-PCR. The genes included are (**A**) APP, (**B**) MAPT, (**C**) NFkB, (**D**) GSK 3α, and (**E**) GSK 3β. All changes in the expression levels were compared to the negative control group (STZ 3 mg/kg) and the negative group (3 mg/kg STZ) was compared to the control group. Data are expressed as Mean ± SEM, n = 4 and statistical analysis by one-way ANOVA followed by Dunnett test **** *p* < 0.0001, ^####^ *p <* 0.0001.

In the hippocampal region, the APP mRNA levels were significantly up-regulated when administered with STZ as compared to the control group, depicted in Figure 4A. Similarly, even the MAPT, NFκB, GSK3α, and GSK3β were observed to be up-regulated when administered with STZ as demonstrated in Figure 4B–E, respectively. This up-regulation was decreased significantly by OS extract treatment as compared with the negative (STZ 3 mg/kg) group. All the five mRNA expression levels, namely APP, MAPT, NFκB, GSK3α, and GSK3β, were observed to be significantly lower when treated with OS extract. The expression of APP mRNA was observed to be down-regulated in all the three doses of OS extract, and similarly, even the expression levels of MAPT, NFκB, GSK3α, and GSK3β mRNA were observed to be decreased in all the three doses of OS extract. 

**Figure 5:**
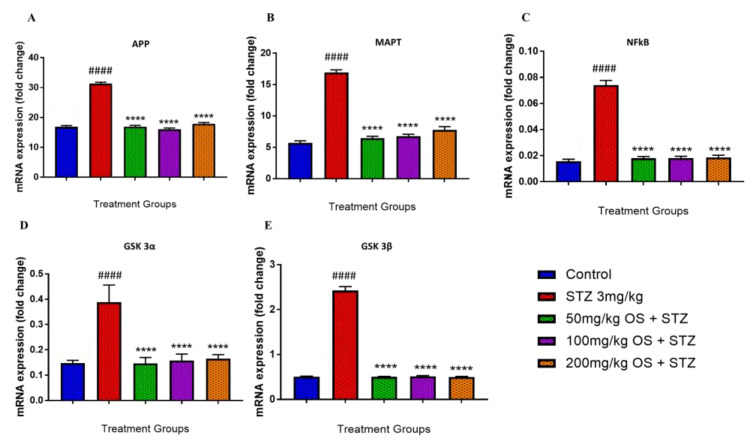
Gene expression in the rat pre-frontal cortical determined by real time-PCR. The genes included are (A) APP, (B) MAPT, (C) NFkB, (**D**) GSK 3α, and (**E**) GSK 3β. All changes in the expression levels were compared to the negative control group (STZ 3 mg/kg) and the negative group (3 mg/kg STZ) was compared to the control group. Data are expressed as Mean ± SEM, n = 4 and statistical analysis by one-way ANOVA followed by Dunnett test **** *p* < 0.0001, ^####^ *p < 0.0001*.

On the other hand, in the pre-frontal cortical region, similar results were also observed, whereby the APP mRNA levels were observed to be significantly augmented when administered with STZ as compared to the control group, as shown in Figure 5A. Likewise, even the MAPT, NFκB, GSK3α, and GSK3β were observed to be increased when administered with STZ as demonstrated in Figure 5B–E, respectively. This up-regulation was decreased significantly by OS extract treatment, whereby all five mRNA expression levels, namely APP, MAPT, NFκB, GSK3α, and GSK3β, were observed to be significantly higher when treated with the OS extract.

## 4. Discussion

The present work aimed at determining if the ethanolic leaf extract of *O. stamineus* has the potential to be a novel treatment for AD. A preliminary dose deciding study to ascertain the therapeutic dose of OS extract was conducted where the LD50 value was found to be more than 2000 mg/kg, and therefore, the 1/20th, 1/10th, and 1/5th was chosen as therapeutic doses, corresponding to a dose range of 100 mg/kg, 200 mg/kg, and 400 mg/kg, respectively. However, when behavioral studies were conducted using these doses, the 400 mg/kg group were found to be exhibiting a neurobehavioral effect on coordination and motor activity and were not able to demonstrate any reliable results. Thus, for this study, a new range of doses was used. The dose of 200 mg/kg was employed as the highest dose, and 50 mg/kg, as well as 100 mg/kg, were used as the low dose and medium dose, respectively. All three doses did not demonstrate any side effects. *O. stamineus* ethanolic extract was employed in this study as ethanolic extracts of *O. stamineus* were found to possess the highest concentration of phenolic compounds, followed by methanolic and aqueous extracts [29] Therefore, since oxidative stress plays a significant role in AD, particularly the phenols in *O. stamineus* such as rosmarinic acid that exert free radical scavenging, anti-inflammatory and antioxidant effects [21,30], ethanolic extract of *O. stamineus* serves as the ideal choice for this experiment.

ICV injection of STZ has been established to be characterized by a progressive decline in learning and memory [31]. In this present study, a dose of 3 mg/kg STZ was employed, which has been shown to not interfere with the changes in the peripheral blood glucose level but induce a significant cognitive impairment in all animals [15,32,33]. Additionally, central administration of low STZ doses triggers an insulin-resistance brain state that produces similar neuropathology and biochemical alterations observed during AD, enabling the pathophysiology of sporadic Alzheimer’s disease (sAD) to be further comprehended. Thus, the expression of increased phosphorylated tau protein in the hippocampus, as well as the accumulation of β amyloid in the meningeal capillaries, suggest that the ICV-STZ model recapitulates most of the sAD pathological feature, and hence, can serve as an apt experimental model of developing the AD hallmarks [13,34].

The performance of animals during the behavioral assessments for spatial memory acquisition and retention using elevated plus maze and passive avoidance test is well documented to estimate the extent of neuronal injury. The present study demonstrated that that treatment with OS extract improved memory retention as evidenced by the improved inflexion ratio observed in the EPM test as well as the increase in the step-through latency observed in the OS treated rats. Previous studies demonstrated significant cognitive impairment in the ICV-STZ treated group [15,35,36,37,38]. In our study, similar results were observed, whereby bilateral ICV administration of STZ resulted in spatial memory deficit, as observed by the decrease in step-through latency in the passive avoidance test and decrease in inflexion ratio observed in the elevated plus-maze test indicating memory impairment. However, when the STZ-injected rats were treated with OS extract, improved performances in both tests were observed. The positive effects of OS extract were evident with the rats that were treated with 50 mg/kg and 100 mg/kg OS extract namely where a decrease in transfer latency was observed in which the rats were able to remember and enter the closed arm quickly compared to the training session, which was observable by the improved inflexion ratio. The rats that were treated with 200 mg/kg did show improved memory retention as compared to the STZ treated group, but not as much as that observed in the 50 mg/kg and 100 mg/kg OS treated group. A ceiling effect was observed with higher doses. Therefore, based on the behavioral analyses, we can conclude that the spatial memory was improved. Thus, OS extract might influence spatial memory retention, which needs to be further explored.

The underlying mechanism for the improvement in memory retention observed in the behavioral studies was further explored by evaluating the biochemical parameters, such as the expression of amyloid precursor protein (APP), microtubule-associated protein tau (MAPT), nuclear factor kappa-light-chain-enhancer of activated B (NFκB), glycogen synthase kinase-3 alpha (GSK3-α), and glycogen synthase kinase 3 beta (GSK3-β) genes in rats treated with OS extract and STZ. Beta-amyloid (Aβ) and tau are some of the key aspects of AD and are undeniably crucial in comprehending the pathogenesis of AD. The AD amyloid cascade hypothesis postulates that the up-regulation of Aβ triggers the pathogenic hyperphosphorylation of tau, which in turn leads to the formation of neurofibrillary tangles (NFTs), thus causing neurodegeneration. Furthermore, dysregulation of GSK3 has been implicated in numerous neurodegenerative diseases, including AD [39,40,41]. Additionally, GSK3 plays a key role in AD as its deregulation accounts for most of the pathological hallmarks of the disease observed in both sporadic and familial AD. Both GSK3β and GSK3α stimulates tau hyper-phosphorylation at both primed and non-primed phosphorylation sites, in both cell culture models, as well as in vitro models of neurodegeneration, further implicating GSK3 as a vital factor in AD [42,43]. GSK3β has been said to influence the abnormal tau hyperphosphorylation, a key component of neurofibrillary tangles observed in the AD brain, which enhances tau aggregation and neurotoxicity [44,45]. On the other hand, GSK3α has been shown to monitor APP cleavage, resulting in the augmented Aβ production [46,47]. Although increased expression of GSK3 is not the main cause of the disease, augmented GSK3 could serve to enhance the production of Aβ, which in turn also trigger tau hyper-phosphorylation and neuronal degeneration in both FAD and sAD, which is in line with the amyloid cascade hypothesis of AD. In the present study, the induction of STZ demonstrated overexpression of all the key genes namely APP, MAPT, GSK3-α, and GSK3-β in both the hippocampus and the prefrontal cortex region. However, when treated with OS extract, the expressions of all these genes were observed to be suppressed indicating maximum protection, and hence, reducing AD pathology.

## 5. Conclusions

In summary, the present study demonstrated that OS extract is effective in ameliorating ICV streptozotocin-induced behavior alterations. Additionally, we also established that the GSK3α-GSK3β pathway could serve as the potential target for beta-amyloid and tau accumulation characteristically observed in AD condition and OS extract could potentially inactivate this pathway, and hence, serve as a promising treatment for neurodegenerative diseases such as Alzheimer’s disease.

## Figures and Tables

**Table 1:** UHPLC-MS small molecules identified in *Orthosiphon Stamineus stamineus* extract.

RT (min)	Mass (m/z)	Compound Identified	DB Formula	Compound Class
0.58	175.9546	Methylselenopyruvate	C4 H6 O3 Se	Oxo carboxylic acid
0.58	150.0317	Piperonal	C8 H6 O3	Benzodioxoles
0.635	103.0993	2-Amino-3-methyl-1-butanol	C5 H13 N O	Valinol
0.648	196.0369	Haematommic Acid	C9 H8 O5	Amides
0.650	360.318	(R)-O-(3,4-Dihydroxycinnamoyl)-3-(3,4- dihydroxyphenyl)lactic acid (Rosmarinic acid)	C18 H16 O8	Flavonoids
0.651	265.1152	D-1-[(3-Carboxypropyl)amino]-1-deoxyfructose	C10 H19 N O7	Carboxylic acid
0.651	404.0875	Asp-Tyr-OH	C18 H16 N2 O9	Amino acid
0.654	309.1058	N-Acetyl-a-neuraminic acid	C11 H19 N O9	Sialic acid (antioxidants)
0.663	117.0789	Valine	C5 H11 N O2	Amino acid
0.673	115.0632	3-Acetamidopropanal	C5 H9 N O2	Monocarboxylic acid amide
0.684	174.0999	Gly Val	C7 H14 N2 O3	Amino acid
0.686	192.0633	Quinic acid	C7 H12 O6	Cyclitol carboxylic acid (plant metabolite)
0.693	232.1057	Asp Val	C9 H16 N2 O5	Amino acid
0.716	125.0477	3-Hydroxyaminophenol	C6 H7 N O2	Phenols
0.717	304.1276	2′-Deoxymugineic acid	C12 H20 N2 O7	Tricarboxylic acid
0.717	135.0546	Adenine	C5 H5 N5	Amino acid
0.753	117.0791	Isoamyl nitrite	C5 H11 N O2	Nitrites
0.758	279.1308	N-(1-Deoxy-1-fructosyl)valine	C11 H21 N O7	Amino acid
0.828	123.0321	Isonicotinic acid	C6 H5 N O2	Carboxylic acid
0.882	208.094	Ethyl beta-D-glucopyranoside	C8 H16 O6	Glucoside
0.883	162.0526	3-Hydroxy-3-methyl-glutaric acid	C6 H10 O5	Carboxylic acid (plant metabolites)
0.886	256.0589	Piscidic Acid	C11 H12 O7	Phenols
0.892	204.0271	Oxaloglutarate	C7 H8 O7	Tricarboxylic acid
0.939	271.1054	Deidaclin	C12 H17 N O6	Glycoside
0.947	174.0162	trans-Aconitate	C6 H6 O6	Carboxylic acid anion (metabolite)
0.947	146.0212	Methyloxaloacetate	C5 H6 O5	Dicarboxylic acid
0.948	192.0271	Citric acid	C6 H8 O7	Tricarboxylic acid
0.956	187.048	1-(Malonylamino)cyclopropanecarboxylic acid	C7 H9 N O5	Carboxylic acid
1.024	189.064	L-2-Amino-6-oxoheptanedioate	C7 H11 N O5	Oxo dicarboxylic acid
1.025	171.053	Tetrahydrodipicolinate	C7 H9 N O4	Dicarboxylic acid anion
1.038	293.1473	N-(1-Deoxy-1-fructosyl)isoleucine	C12 H23 N O7	Amino acid
1.074	131.0945	N,N-Diethylglycine	C6 H13 N O2	Amino acid
1.222	100.0164	Succinic anhydride	C4 H4 O3	Tetrahydrofurandione
1.223	118.0263	Erythrono-1,4-lactone	C4 H6 O4	Lactone (butan-4-olide)
1.381	131.0944	L-Leucine	C6 H13 N O2	Amino acid
1.403	283.0918	8-hydroxy-2′-deoxy Guanosine	C10 H13 N5 O5	Nucleoside
1.452	127.0631	Guvacine	C6 H9 N O2	Amino acid
1.453	145.074	Isobutyrylglycine	C6 H11 N O3	Carboxylic acid (N-acylglycine)
2.075	327.1319	N-(1-Deoxy-1-fructosyl)phenylalanine	C15 H21 N O7	Monosaccharide derivative
2.176	165.0784	Gentiatibetine	C9 H11 N O2	Alkaloids
3.004	198.0526	2-Hydroxy-3,4-dimethoxybenzoic Acid	C9 H10 O5	Phenolic acid
3.005	152.0465	p-Anisic acid	C8 H8 O3	Phenolic acid
5.57	162.0313	3-Hydroxycoumarin	C9 H6 O3	Coumarin
7.663	712.2231	Isoliquiritigenin 4′-O-glucoside 4-O-apiofuranosyl-(1′′′->2′′′)-glucoside	C32 H40 O18	Flavonoids
7.742	684.1694	Cosmosiin Hexaacetate	C33 H32 O16	Phenols
7.802	180.0416	4-Hydroxyphenylpyruvic acid	C9 H8 O4	Carboxylic acid (oxo carboxylic acid)
7.923	206.1302	2-Phenylethyl 3-methylbutanoate	C13 H18 O2	Carboxylic ester
7.944	656.1391	Quercetagetin 4′-methyl ether 7-(6-(E)-caffeylglucoside)	C31 H28 O16	Flavonoids
7.993	517.1616	Piperacillin	C23 H27 N5 O7S	Penicillin
8.013	594.1598	Luteolin 7-rhamnosyl(1->6)galactoside	C27 H30 O15	Flavonoids
8.442	226.1203	12-hydroxyjasmonic acid	C12 H18 O4	Oxo carboxylic acid
8.467	608.1184	Prodelphinidin A1	C30 H24 O14	Flavonoids
8.538	206.1301	2-Phenylethyl 3-methylbutanoate	C13 H18 O2	Carboxylic ester
8.582	596.1388	Quercetin 3-alpha-arabinopyranosyl-(1->2)-glucoside	C26 H28 O16	Flavonoid glycoside
8.612	448.1012	6-Hydroxyluteolin 7-rhamnoside	C21 H20 O11	Flavonoids
8.702	464.0979	Robinetin 7-glucoside	C21 H20 O12	Flavonoids
8.985	464.096	5,6,7,3′,4′-Pentahydroxy-8-methoxyflavone 7-apioside	C21 H20 O12	Flavonoids
9.062	294.0376	Tricrozarin A	C13 H10 O8	Quinone
9.316	196.1096	4-(2-hydroxypropoxy)-3,5-dimethyl-Phenol	C11 H16 O3	Phenols
9.339	448.1013	6-Hydroxyluteolin 5-rhamnoside	C21 H20 O11	Flavonoids
9.365	720.1688	Xanthochymuside	C36 H32 O16	Flavonoids
9.612	520.1583	5-Hydroxy-7,8,2′,3′-tetramethoxyflavone 5-glucoside	C25 H28 O12	Flavonoids
9.733	342.0744	Iriskumaonin	C18 H14 O7	Flavonoids
9.734	162.0318	3-Hydroxycoumarin	C9 H6 O3	Coumarins
9.734	180.0424	4-Hydroxyphenylpyruvic acid	C9 H8 O4	Phenols
9.823	520.1575	Quercetin 5,7,3′,4′-tetramethyl ether 3-galactoside	C25 H28 O12	Flavonoids
10.028	538.1122	Lithospermic acid	C27 H22 O12	Benzofuran
10.028	718.1537	Salvianolic acid L	C36 H30 O16	Stilbenoids
10.352	506.143	Morin 3,7,4′-trimethyl ether 2′-glucoside	C24 H26 O12	Flavonoids
10.441	520.1012	Melitric acid B	C27 H20 O11	Cinnamic acids
10.444	538.1113	Melitric acid A	C27 H22 O12	Cinnamic acids
10.996	208.0736	2,5-Dimethoxycinnamic acid	C11 H12 O4	Cinnamic acids
11.032	330.0741	Hypolaetin 8,3′-dimethyl ether	C17 H14 O7	Flavonoids
11.246	254.1879	Kikkanol A	C15 H26 O3	Sesquiterpenoid
11.55	358.1057	Corymbosin	C19 H18 O7	Flavonoids
11.664	374.1366	(2S)-5,6,7,3′,4′-Pentamethoxyflavanone	C20 H22 O7	Flavonoids
11.783	328.0949	Luteolin 7,3′,4′-trimethyl ether	C18 H16 O6	Flavonoids
12.256	342.1106	5,7-Dihydroxy-3′,4′-dimethoxy-6,8-dimethylflavone	C19 H18 O6	Flavonoids
12.306	272.2345	16-hydroxy hexadecanoic acid	C16 H32 O3	Juniperic acid
12.42	180.1145	3-tert-Butyl-5-methylcatechol	C11 H16 O2	Phenols
12.465	372.1213	7,8,3′,4′,5′-Pentamethoxyflavone	C20 H20 O7	Flavnonoids
12.706	344.09	Wightin	C18 H16 O7	Flavonoids
12.764	314.0793	Luteolin 5,3′-dimethyl ether	C17 H14 O6	Flavonoids
13.198	342.1108	5,7,2′,5′-tetramethoxyflavone	C19 H18 O6	Flavonoids
13.673	310.1784	methyl 8-[2-(2-formyl-vinyl)-3-hydroxy-5-oxo-cyclopentyl]-octanoate	C17 H26 O5	Long chain fatty acid
14.622	328.0942	Luteolin 7,3′,4′-trimethyl ether	C18 H16 O6	Flavonoids
21.342	390.2771	3α,12α-Dihydroxy-5β-chol-8(14)-en-24-oic Acid	C24 H38 O4	Cholanoids
